# Accessory mitral valve tissue with mitral complex structural abnormality

**DOI:** 10.1007/s12574-015-0245-5

**Published:** 2015-05-09

**Authors:** Koichiro Imai, Mariko Kawata, Hiroyuki Okura, Shiro Uemura

**Affiliations:** Department of Cardiology, Kawasaki Medical School, 577 Matsushima, Kurashiki, Okayama 701-0192 Japan

## Images in cardiovascular ultrasound

**Fig. 1:**
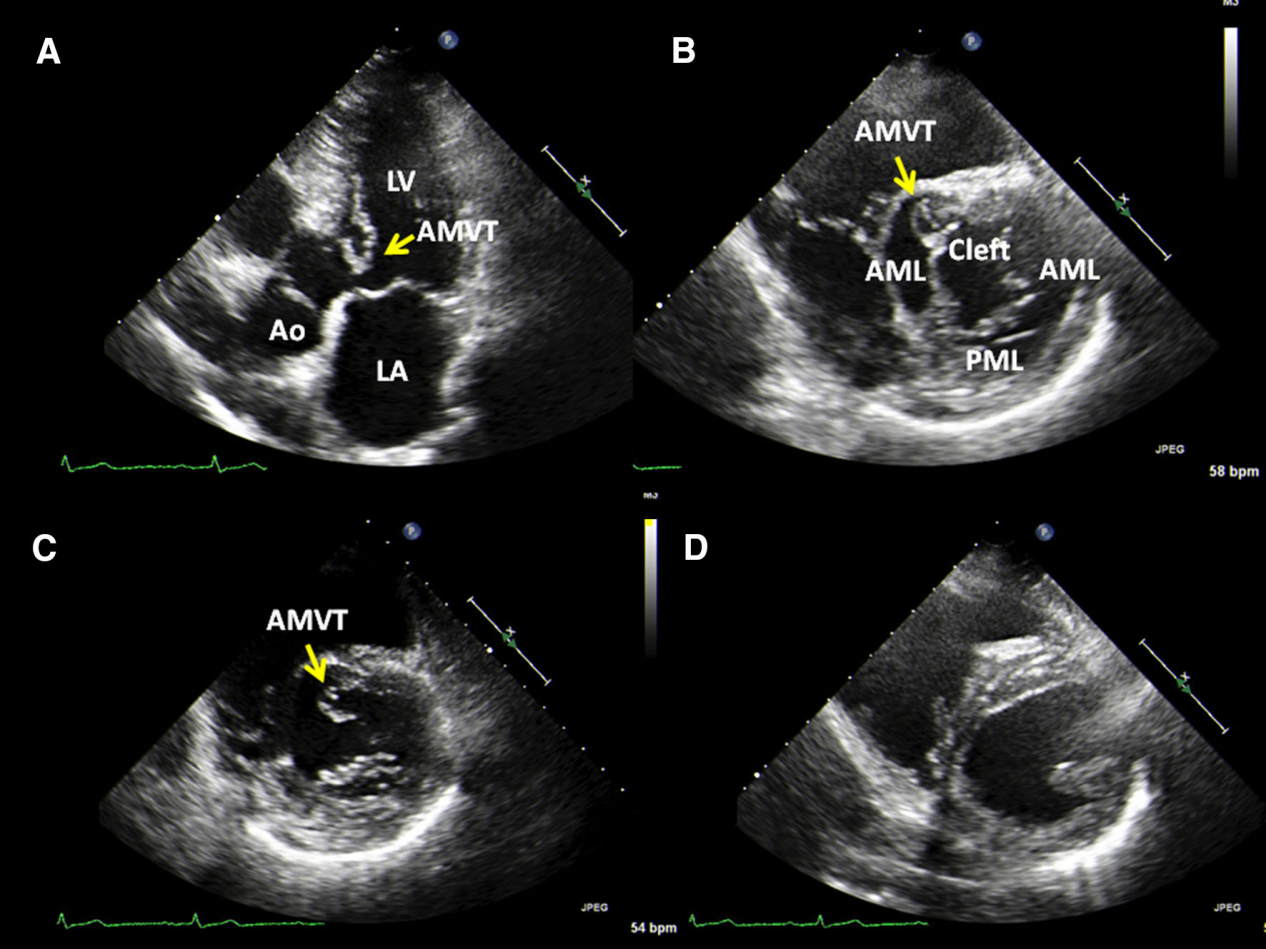
**a** Transthoracic echocardiography in the apical long-axis view shows accessory mitral valve tissue (AMVT) (*arrow*) into the left ventricular outflow tract (LVOT). *LV* left ventricle, *LA* left atrium, *Ao* aorta, *AMVT* accessory mitral valve tissue. **b** Transthoracic echocardiography in the parasternal short-axis view in diastole shows mitral valve leaflet cleft and AMVT (*arrow*) attached from the base of the intraventricular septum to anterior mitral leaflet. *AML* anterior mitral leaflet, *PML* posterior mitral leaflet. **c** Transthoracic echocardiography in the parasternal short-axis view in systole. **d** Transthoracic echocardiography in the parasternal short-axis view shows single papillary muscle (loss of posterior papillary muscle)

**Fig. 2:**
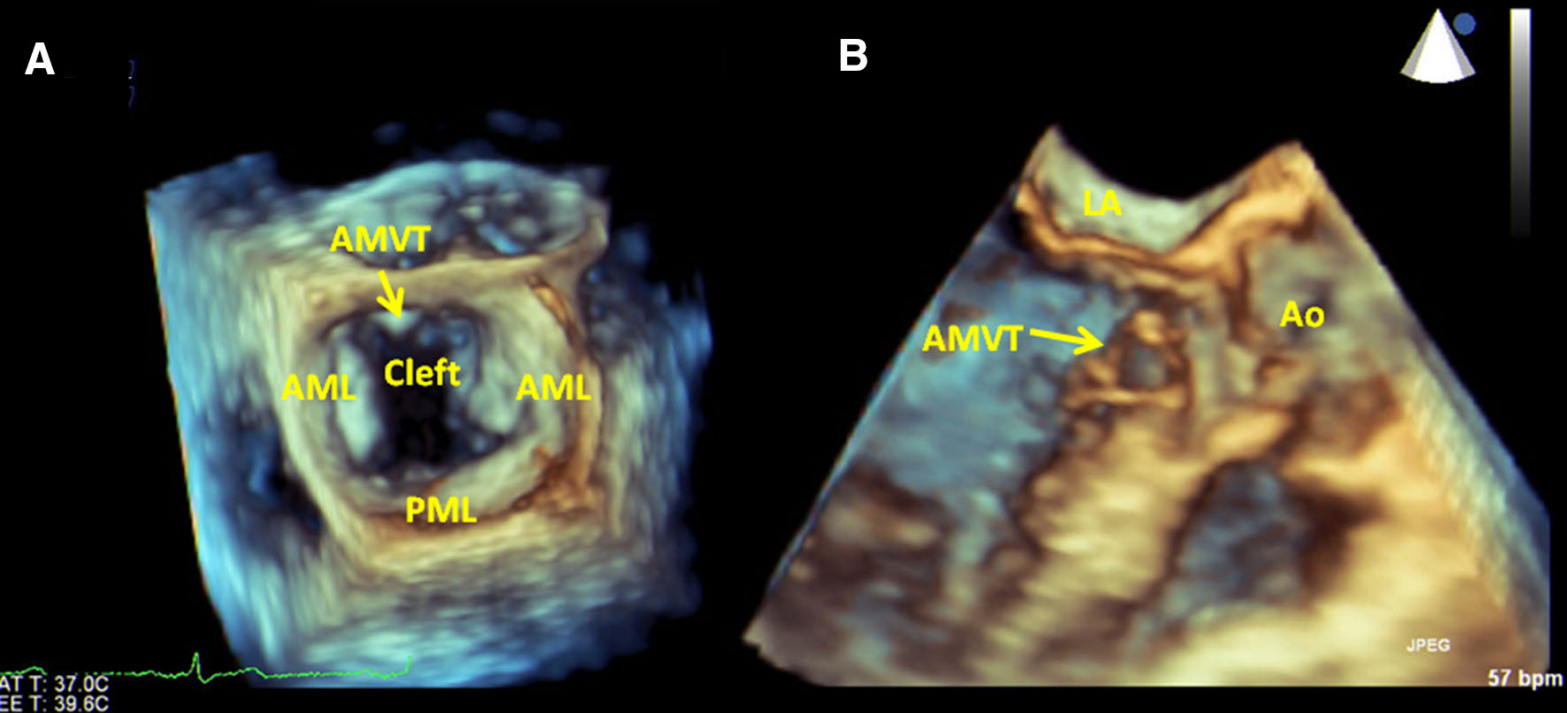
**a** Three-dimensional transesophageal echocardiography shows in diastole show mitral valve leaflet cleft and AMVT (*arrow*) attached from base of intraventricular septum to anterior mitral leaflet. **b** Three-dimensional transesophageal echocardiography in the long-axis view shows accessory mitral valve tissue (AMVT) (*arrow*) into the LVOT

A 64-year-old Japanese man was referred to our hospital because of palpitation. An electrocardiogram revealed first-degree atrioventricular block without ST-T change. An echocardiography showed structural abnormality into the left ventricular outflow tract (LVOT), mitral valve leaflet cleft without mitral regurgitation, and single papillary muscle (loss of posterior papillary muscle) (Figs. 1, 2). A structural abnormality was attached between the base of the intraventricular septum and anterior mitral leaflet, which was mobile in the cardiac cycle. Peak velocity of LVOT was 1.7 m/s. Cardiac systolic function was normal. We diagnosed accessory mitral valve tissue (AMVT) with mitral complex structural abnormality (mitral valve leaflet cleft and single papillary muscle).

AMVT is a rare congenital cardiac malformation. The age of AMVT diagnosis ranged from newborns to 77 years [1]. It was reported that the incidence of AMVT in adults was 1/26,000 echocardiogram [2]. Although the exact embryologic mechanism of AMVT formation is not clear, it may stem from the abnormal or incomplete separation of the mitral valve from the endocardial cushions [3]. AMVT is associated with other congenital intracardiac and vascular malformations such as ventricular septal defect [4]. In this case, we found AMVT with a combination of mitral valve leaflet cleft and single papillary muscle (loss of posterior papillary muscle). To our knowledge, this is the first reported case of AMVT with a combination of mitral valve leaflet cleft and single papillary muscle.

An echocardiography can be considered the gold standard modality for the diagnosis of AMVT with other cardiac abnormality.

Although single papillary muscle usually combined the parachute mitral valve, the parachute mitral valve was not observed in this case. The majority of patients with AMVT have no symptoms, such as chest pain, palpitation, and syncope [5–7]. However, this patient was referred to our hospital because of palpitation. No arrhythmia was observed in electrocardiogram monitoring.
